# A qualitative examination of inappropriate hospital admissions and lengths of stay

**DOI:** 10.1186/1472-6963-9-44

**Published:** 2009-03-05

**Authors:** Christina L Hammond, Lorraine L Pinnington, Margaret F Phillips

**Affiliations:** 1 Rehabilitation Research and Education Group, School of Community Health Sciences, University of Nottingham, Derby, UK; 2 Rehabilitation Research and Education Group, School of Graduate Entry Medicine & Health, University of Nottingham, Derby, UK

## Abstract

**Background:**

Research has shown that a number of patients, with a variety of diagnoses, are admitted to hospital when it is not essential and can remain in hospital unnecessarily. To date, research in this area has been primarily quantitative. The purpose of this study was to explore the perceived causes of inappropriate or prolonged lengths of stay and focuses on a specific population (i.e., patients with long term neurological conditions). We also wanted to identify interventions which might avoid admission or expedite discharge as periods of hospitalisation pose particular risks for this group.

**Methods:**

Two focus groups were conducted with a convenience sample of eight primary and secondary care clinicians working in the Derbyshire area. Data were analysed using a thematic content approach.

**Results:**

The participants identified a number of key causes of inappropriate admissions and lengths of stay, including: the limited capacity of health and social care resources; poor communication between primary and secondary care clinicians and the cautiousness of clinicians who manage patients in community settings. The participants also suggested a number of strategies that may prevent inappropriate admissions or reduce length of stay (LoS), including: the introduction of new sub-acute care facilities; the introduction of auxiliary nurses to support specialist nursing staff and patient held summaries of specialist consultations.

**Conclusion:**

Clinicians in both the secondary and primary care sectors acknowledged that some admissions were unnecessary and some patients remain in hospital for a prolonged period. These events were attributed to problems with the current capacity or structuring of services. It was noted, for example, that there is a shortage of appropriate therapeutic services and that the distribution of beds between community and sub-acute care should be reviewed.

## Background

A number of patients, both in the UK and worldwide, are admitted to acute hospitals when it is not medically appropriate [1-5]. Similarly, a large proportion of in-patient stays are inappropriate [3,5,6]. Inappropriate admissions and delayed discharges are relatively high on the research and policy agenda due to concerns about bed pressures, hospital acquired infections and a drive to control increasing healthcare costs. The National Service Framework for Long Term (Neurological) Conditions [7], has highlighted similar concerns, particularly as the prevalence of people with Long Term Neurological Conditions (LTNCs) is increasing.

Approximately, 20% of people admitted to an acute hospital have a LTNC [7]. These patients frequently have specialised needs, they are often regular users of secondary care services and they are often reliant on the provision of community health and social care services. Therefore, when an exacerbation occurs, it can be difficult to avoid an inappropriate admission, particularly if services need to be delivered more frequently. Also, if specialist care needs are not met whilst the patient is in hospital, secondary complications, such as pressure sores and contractures can occur and these can delay discharge [7].

Studies that examine the appropriateness of admissions and lengths of stay, often strive to highlight the causes underlying the event or to identify predictive factors. However, very few previous studies have sought to explore these issues in-depth. As inappropriate admissions and LoS are complex phenomena, it can be difficult to uncover causative or contributory factors. Similarly, if quantitative methods are used alone, potential solutions cannot be explored in detail [8]. Criticisms have sometimes been levied at the NHS for failing to appreciate how factors interrelate within the system. Consequently, service providers and commissioners can fail to appreciate the wider picture and may not recognise how change in one service can have a significant impact on another aspect of the system [9]. It is clear, for example, that changes in the provision of out of hours services have led to concerns in the UK that deputising locums (who are unlikely to be familiar with the patient's medical or social history) might refer patients to another service or hospital more often than their counterparts, particularly if the patient has a LTC [10,11]. Similarly, general practitioners' referral practices may be influenced by patient pressure or fear of litigation and ultimately this could have an impact on the appropriateness of hospital referrals [12,13]. To date, these issues have not been explored in depth and nor has their impact on the appropriateness of admissions or LoS.

In summary, no previous studies have focussed solely on the appropriateness of admission or LoS for patients with LTNCs. We conducted a quantitative study prior to this work, which examined the appropriateness of acute admissions and LoS of patients admitted to one large acute trust (namely, Derby Hospitals NHS Foundation Trust) and this revealed that inappropriate admissions and LoS were occurring amongst patients with LTNCs. With the increasing specialist needs of those with LTNCs and the increased use of primary and secondary care services, solutions tailored specifically to the needs of such patients are required. Clinicians who practice in the NHS daily can provide an invaluable insight into the immediate and wider health care system and its associated problems, and are well placed to identify perceived causes and practical solutions. The primary purpose of this follow-up qualitative study was to address the methodological weaknesses of previous studies by (a) examining, in depth, the perceived causes of inappropriate admissions and lengths of stay and (b) highlighting practical solutions or strategic changes which might alleviate the problem.

## Methods

### Participants

Eight clinicians were recruited to take part in two focus groups (three men and five women). In order to ensure that clinicians were familiar with the functioning of the Trust and services available locally, only participants who had been employed by Derby Hospitals NHS Foundation Trust for a minimum period of a year were recruited. Clinicians representing: respiratory medicine (n = 1), neurology (n = 4), general practice [GP] (n = 1), care of the elderly (n = 1) and acute medicine (n = 1) were selected purposefully. Eligible clinicians were emailed a copy of the study information sheet and invited to take part in the research. In order to maximise recruitment prospective participants (n = 15) were approached on several occasions. The sample derived included representatives from all relevant areas and was adequate to meet the study objectives.

The first focus group consisted of a GP (ID1), nurse consultant for older people (ID2), acute care charge nurse (ID3) and a consultant in respiratory medicine (ID4). The second group comprised a community based specialist neurological nurse (ID5), a community based epilepsy nurse (ID6), a consultant neurologist (ID7) and a Parkinson's disease specialist nurse (ID8), see Table 1 for details of the participants and their IDs.

**Table 1:** Participant ID

**ID**	**Occupation**	**Service area**
1	General practitioner	Primary care
2	Older persons nurse	Secondary care
3	Acute care nurse	Secondary care
4	Consultant in respiratory medicine	Secondary care
5	Specialist neurology nurse	Primary care
6	Specialist epilepsy nurse	Primary care
7	Consultant in neurology	Secondary care
8	Specialist Parkinson's disease nurse	Secondary care

### Setting

Participants were recruited from the Derby Hospitals NHS Foundation Trust or Derbyshire County Primary Care Trust (PCT). The Derby Hospitals NHS Foundation Trust includes two acute hospitals with 1,157 beds (56 of which are medical assessment beds and 352 general medical beds) and the PCT provides a range of health and social services. Both serve a combined population of approximately 600,000 people.

### Data collection

Prior to the focus group meeting participants were given a brief schedule of topics and asked to: (a) consider the perceived causes of and issues relating to inappropriate admissions and/or LoS; (b) identify possible interventions to reduce the incidence of inappropriate admissions and/or LoS; (c) identify mechanisms that might facilitate or impede implementation of new or modified strategies. The schedule was sufficiently flexible to allow issues that were relevant to the topic to be discussed. The meetings were facilitated by CH. Each participant was asked in turn to describe what they perceived were the causes of inappropriate admissions/lengths of stay. Once each of the participants had provided their comments the group were given an opportunity to discuss the causes/issues raised. This process was repeated for the second question: what can be done to reduce the number of patients who experience an inappropriate admission or LoS. When discussing suggested strategies, participants were asked specifically to identify factors which could delay, complicate or prevent implementation. Focus group meetings were recorded digitally with the permission of each participant and transcribed verbatim.

### Analysis

Focus group transcripts were analysed using the method of thematic content analysis [14]. Thematic content analysis involves an initial reading of the transcript in order to familiarise the researcher with the data, during which points of interest are noted. During a second reading of the transcript data are summarised and the text is assigned to analytical categories, often referred to as themes. An understanding of the meaning of themes/categories is then produced [15]. In order to validate the results, the themes which are produced are checked against the original data to ensure they reflect the data collected. All those who participated were given an opportunity to review the results and gave permission for anonymised quotes to be included in any reports or presentations arising from the study. The methods were approved by the Derby National Research Ethics Committee (NREC).

## Results

The findings of the focus groups discussion examine the causes of inappropriate admissions and lengths of stay and possible interventions to reduce the occurrence of such admissions/lengths of stay. Five causes of inappropriate admissions and five potential interventions to reduce inappropriate admissions/lengths of stay were identified by participants.

### Causes of inappropriate admissions and inappropriate length of stay

Many of the themes discussed by the focus groups were interrelated, however, the issues focused primarily on the following broad themes: a lack of health and social care resources, the admitting (generalist) clinician's lack of knowledge of the patient or the condition; communication difficulties between primary and secondary care clinicians; patient preferences; the perceived benefits of admission to hospital.

### Health and social care services

Structural problems, namely a lack of health and social care resources in the community, were thought to be one of the main causes of both inappropriate admissions and LoS. This was perhaps one of the most pervasive concerns of the clinicians, both in the multi-disciplinary and neurology focus group. More specifically, it was noted that few resources can be accessed urgently (i.e., within a day), and those that exist have limited capacity. Some of the secondary problems associated with LTNCs (e.g. ventilatory muscle weakness) can cause rapid deterioration if patients become ill and a delay occurs before treatment is instigated:

"If you've got somebody who is on the brink, yes they don't need acute services but they could tip very easily.....You would need a quick response resource available to us" (ID2).

The participants acknowledged, therefore, that for some patients admission to an acute hospital is the only option available if other services cannot be accessed rapidly.

Patients with LTNCs frequently need health and social care services to be provided in the community following discharge. A perceived barrier to discharge related to the limited availability of community health and social care resources, particularly physiotherapy and occupational therapy. Also, participants commented that it is often difficult to secure convalescent nursing care for patients who no longer require acute medical and nursing and this frequently results in protracted lengths of stay. Similarly, the availability of rehabilitation beds was perceived to be limited as there is a poor uptake/allocation of community hospital beds by and to GPs.

Another specific resource limitation the participants identified was specialist nursing posts. Specialist nurses were thought to play a fundamental role in the care of patients with LTNCs and their interventions were thought to minimise unnecessary hospital admissions and to facilitate the discharge of patients with LTNCs from hospital. Irrespective of this, the group felt that the number of specialist nursing posts was often reduced during rationalisation. Participant ID 7, a hospital based neurological consultant commented:

"The first places that were hit was specialist nurses... talking about shooting yourself in the foot, this is unbelievable because this costs more... ..it's specialist nurses who made the whole system tick." (ID7)

In addition to limited community resources, participants in the focus group with primarily neurological backgrounds identified several limitations of inpatient services. Within the Trust neurology cover was noted to be restricted to four days per week. Therefore, if a patient is admitted to a Derby hospital with a neurological complaint that is deemed to be critical on a day when no specialist neurological cover is available patients are transferred to Nottingham University Hospitals NHS Trust (NUH). However, those who do not need to be transferred, but still require specialist neurological advice, need to wait until they can be reviewed by a neurologist locally and this can delay discharge. Such concerns were not reiterated by the multi-disciplinary focus group and therefore they may be unique to the neurologists.

### Admitting clinicians lack of knowledge of the patient or the condition

The decision making processes admitting clinicians use were thought to have a direct impact on the appropriateness of some admissions and was a considerable concern for all participants. Clinicians who were not familiar with the patient (such as 'out of hours staff'), were thought to admit many patients to hospital inappropriately. This conservative approach was thought to be attributable to the fact that visiting clinicians were unfamiliar with the patient's baseline state of health and were therefore more cautious. Participants were sympathetic to the situation faced by admitting clinicians and explained that people with LTNCs frequently have physical and mental impairments, which can fluctuate and vary in type and severity. Clinicians who are unfamiliar with the patient may mistakenly interpret the patient's problems as acute rather than chronic, and therefore choose to admit patients to hospital:

"You've got clinicians who, although are highly trained, don't particularly know the patient and don't particularly know the circumstances and have to make an on the spot decision based on what's in front of them......and therefore make quite appropriate decisions in terms of the clinical illness they see, but don't know the background and don't know what the patients have coped with before." (ID 2).

The management of patients with Parkinson's disease was perceived as being particularly poor in the community by those in the neurology focus group only. There was a perceived lack of knowledge amongst admitting clinicians regarding the effective community management of such patients. As a result, patients with Parkinson's disease, were thought to experience inappropriate admissions above and beyond those experienced by other condition groups.

### Communication difficulties between primary and secondary care

The type, level and responsiveness of communication between departments and key individuals were thought to have a significant impact on the timeliness and outcome of many hospital discharges. Poor communication did not appear to be a mutual problem; rather it appeared that those in secondary care infrequently sought to communicate with those in primary care despite the efforts of primary care clinicians to do so. Clinicians who were involved in the day to day care of patients noted that they were rarely informed when a patient was admitted to hospital:

"There's never any communication backwards of when someone's been admitted and why they've been admitted.......Because it is always us and them. We're in primary care, you're in secondary care and never the twain shall meet." (ID 6).

Similarly, some primary care employees felt that if they could share their knowledge and have some input into the patient's management whilst in hospital, this could expedite discharge. In their experience, however, such inter-agency communication occurred rarely. It was suggested that more use could be made of automatic alert systems which enable clinicians to be informed when a patient is admitted to hospital. One participant in the group was using such a system and felt it should be adopted more widely.

### Patient preferences

Patients' preferences for care were thought to be key determining factors in the type of care that was provided by one individual (ID 1). In some cases, it was felt that patients who expressed a wish to be admitted could be admitted to hospital when no acute care was required. From a general practice perspective these preferences were sometimes motivated by the individual's or the family's desire to control costs i.e., to avoid or minimise the fees associated with respite or long term care (which was often a more appropriate form of care than hospital admission):

"The other thing is that the patients don't really like being admitted to residential or nursing homes by us [GPs] because of the cost implications" (ID1).

There was little consensus regarding this as a cause of inappropriate admissions amongst the multi-disciplinary focus group. Also it was not raised as a concern by those in the neurology (uni-disciplinary) focus group however this may be due to their inexperience of admitting patients from the community to hospital.

### The benefits of admission to hospital

Whilst participants were in agreement concerning the causes of inappropriate admissions and lengths of stay, they expressed different opinions when asked to consider the seriousness of inappropriate admissions. One participant (an acute care clinician) (ID3) believed that although some hospital admissions were inappropriate on medical grounds they often enabled a problem to be resolved quickly:

"I sometimes think that perhaps we ought to look at it from the other way around... Bring them into to hospital, sort them out as best you can, but then make the discharge of the patients a bit quicker." (ID 3).

This point was reinforced by the GP:

"Yes it's very reassuring for us [GPs] even if somebody has been in for two or three days, you know they've been checked over." (ID 1).

However, a respiratory consultant physician highlighted that whilst an 'inappropriate admission' may have these unintentional benefits, they often led to prolonged hospital stays:

"The only problem with getting people in sometimes is that ....sometimes when you get patients in it can be a big problem getting them out." (ID 4).

There was often disagreement between clinicians about what care should be provided in an acute setting and what should be provided in a community setting.

### Strategies to prevent inappropriate admissions and inappropriate lengths of stay

Once participants had identified the perceived causes of inappropriate admissions and inappropriate lengths of stay they went on to identify strategies that may reduce the frequency or impact of the underlying causes. Suggestions typically sought to improve communication of primary and secondary clinicians, specialist knowledge, and structural inefficiencies. Participants identified five interventions which included: education sessions, summaries of specialist consultations retained in the patients' home, letters advising GPs of the appropriateness of future admissions and the provision of services including the introduction of sub acute services and auxiliary support for specialist nurses.

### Education sessions for GPs

As outlined above, it was felt that GPs and out of hours staff often had a relatively limited exposure to and thus knowledge of LTNCs. Providing opportunities to increase or update training in the management of neurological conditions was recommended as one method by which inappropriate admissions might be avoided. One participant (ID2) commented, however, that this type of post-registration education was already occurring and available to 'out of hours' staff and in their view, the issue of appropriateness was already a high priority. In terms of GP education, concerns were raised about how to target and incentivise those who would benefit most from education. The participants felt that in their experience the GPs with the greatest need for training in this field were least likely to attend educational sessions. It was suggested that targeting GPs with high numbers of patients with LTNCs, or where there were high admissions of such patients may be effective. In practical terms GP were noted to have specific time dedicated to continued learning and it was felt that this time would allow GPs to attend an education session/s.

### Summaries of specialist consultations

It was felt that many patients were admitted inappropriately because 'out of hours' clinicians were unfamiliar with the patient's condition and current health state. Written management plans, such as those being developed currently by community matrons in the area, were thought to be an effective means of reducing inappropriate admissions. It was suggested that for patients with a LTNC information regarding the outcomes of specialist consultations would help to ensure that clinical decision making by out of hours staff was based on more detailed and patient specific information..

"One thing we could do as clinicians is just copy letters of erm, letters we write to GPs, for patients with very difficult or specialist conditions....Because what happens sometimes when you are assessing a patient, the diagnosis is not clear, you don't know what the patient has been like...it is....very useful for whoever is assessing them....If you have four letters... telling you this patient has been very stable its very different from four letters saying the patient is clearly declining." (ID 4).

In practical terms it was suggested that these letters could be produced simultaneously i.e., when GP letters were produced and this would obviate the need for additional work or costs. Concerns were raised, however, as to how such letters would be integrated with existing records (e.g. district nursing/community matron records) and how confidentiality would be maintained in situations where patients were unwilling to disclose information regarding their condition, for example, to other members of the household.

### Appropriateness of future admissions

Providing GPs with feedback about the appropriateness of each admission was suggested as a potential strategy to reduce future inappropriate admissions. Clinicians caring for patients in the community were rarely made aware when a patient had been admitted or of the outcome of the admission. Feedback regarding the appropriateness of an admission and whether the patient required future admission or not if the complaint were to reoccur would educate GPs and/or nursing home staff as to when admission was or was not necessary for a specific patient:

"When the patient had actually been seen and assessed and was ready for going, if they thought the admission was inappropriate, for want of a better word, a letter was actually sent out with the patient, especially when they were in a nursing home, saying that further admission was not really appropriate.you... know, there was nothing further we could do for this patient from a, a medical point of view." (ID 3).

However, one participant (ID1, GP) explained how hospital clinicians were often unaware of the admitting circumstances and would frequently have limited information to base their decision of appropriateness on, further highlighting the communication problems between primary and secondary care providers. It was felt that such a letter may not be received gladly by the GP community and could be viewed as a criticism of their medical expertise. The time it would involve to complete the letter could also act as a barrier. The letter would need to be sufficiently detailed for the judgement to be justified yet brief enough to minimise the time it would take to complete. It was also felt that as patients frequently 'push' for admission, if they were to be informed that similar admissions may not be recommended; this might cause some stress and frustration. On the whole, this suggestion was not received enthusiastically by the members of the multi-disciplinary focus group.

### Sub acute facilities

A paucity of sub-acute services often resulted in delayed discharges. It was suggested, therefore, that beds could be allocated to provide 'sub acute' care, in conjunction with relevant care pathways. The pathway could depict at what stage of the admission a patient's care should transfer to a 'sub acute' facility and this would allow patients to be 'stepped down' to a less resource intensive environment:

"There are a number of patients who could be stepped down, not for rehab, but for their ongoing clinical management" (ID 2).

It was suggested that nursing homes and three community hospitals in Derbyshire may be suitable environments to provide sub acute care. It was noted that community hospitals beds were currently assigned to rehabilitation and as a consequence patients with little or no rehabilitation potential were refused access to these beds. Participants emphasised that due to the specific needs of patients with LTNCs it would be essential to ensure that staff were trained appropriately and equipped to deal with the needs of such patients.

In order to ensure that providers of sub acute care were supported adequately community matrons and neurology clinicians were suggested as potential sources of support. Participants believed there would be a large demand for sub acute services and that to cope with the predicted level of demand eligibility criteria would need to be specified and adhered to closely to ensure patients who would benefit most received the service. Specifically the eligibility criteria would need to ensure the service catered for patients who required short term care to enable them to recuperate rather than patients who required long term care and would be unlikely to return to their original place of residence. The overall consensus of the multi-disciplinary group was that this would be a worthwhile and effective service development.

### Specialist nurse support

To maximise the impact and efficiency of specialist neurological nursing services, participants suggested that auxiliary nurses (now referred to as health care assistants), could be trained to provide a basic level of care to patients with LTNCs. Health care assistants it was felt, could care for stable patients thus allowing specialist nurses to concentrate their (limited) resources towards patients with unstable needs or circumstances:

"...giving all the nurse specialists one or two auxiliary nurses to work with them so that...the patients who were stable and just needed monitoring could be monitored. They [nurse specialists] could deal with the very high risk patient. They could also free up time to go into things like residential care, nursing homes, monitor some of those patients and do more of their proactive work...." (ID 2).

The specific training needs of health care assistants were thought to include: knowledge concerning suitable positioning, feeding, swallowing and hydration. However, it was acknowledged that such a service would involve considerable resources and members of the multi-disciplinary group were therefore pessimistic that such a service would be adopted.

## Discussion

These findings provide a detailed insight into the convergent and sometimes divergent perspectives of neurology and multi-disciplinary clinicians concerning the potential causes of inappropriate admissions and lengths of stay. The findings from the second focus group (uni-disciplinary) were consistent with those of the first focus group (multi-disciplinary), therefore demonstrating that data saturation was achieved.

The clinicians involved in this study demonstrated that there is an awareness of, and a concern regarding, inappropriate admissions and delayed discharge.

The problems identified are interrelated and focus mainly on systemic issues with many identifying a lack of services and poor communication of information between clinicians and different agencies as a problem. Unsurprisingly, participants felt there were limitations in the provision of community health and social care resources, particularly community physiotherapy and occupational therapy, which were thought to lead to both inappropriate admissions and delayed discharges. This is consistent with the findings that rehabilitation services are limited and can impact on both hospital admissions and discharges [8]. Structural limitations in the availability and delivery of services will not be unique to the Derbyshire area. Whilst clinicians may seek to ensure patients receive the most appropriate care in the most appropriate setting, clinicians are constrained practically by the services that are available to them. The importance of community and specialist rehabilitation and support has been highlighted in quality requirement five of the long term conditions NSF and should be an area that commissioners seek to address, both in the study locality, Derbyshire, and the rest of the UK. There are two issues here – one is the availability of individual therapy services, and the other is the availability of specialist rehabilitation services. The latter will also have a major impact on communication and coordination for patients, as well as actual resource availability.

Whilst there is an awareness of the lack of rehabilitation service provision, little attention is given to the sub-acute needs of those with little or no rehabilitation potential. A number of nursing-led inpatient units and nursing homes have been introduced. These enable patients who have completed their acute care to be managed by nurses until they are ready for discharge, however prioritise therapeutic or rehabilitative nursing care needs of post-acute patients rather than convalescent needs [16]. The needs of those requiring convalescence care only therefore appear neglected. As suggested earlier, beds allocated specifically to sub-acute care rather than rehabilitative or therapeutic care would enable a number of LTNC patients to be moved from the acute setting whilst they convalesce. Employing a utilization review may be one way of determining the proportion and numbers of beds in acute, sub-acute and rehabilitative care, as has been suggested previously in both Canada and Australia [17,18]

Perhaps the greatest concern is the lack of communication between primary and secondary care clinicians and access to specialist knowledge. The participants acknowledged there was a great need for specialist knowledge when caring for patients with LTNCs, advocating specialist nurses. It is unclear if perceived rationalisation of specialist nursing posts is specific to Derby; however, consideration needs to be given as to how this is impacting on the health care system. The patients of specialist nurses are frequently heavy users of secondary and primary care services. Prevention of admission and assistance in discharging by specialist nurses may in the long run prevent further functional deterioration and therefore inflated use of services. Any immediate savings may therefore be lost in the medium to longer term. The participants' focus on specialist nurses in this way is itself interesting – whilst they can play an important role which could be assisted by health care assistants, the comments may also reveal a lack of awareness of how other disciplines, or the combined resources of such disciplines in the form of a multidisciplinary team, can also have a role.

This study has confirmed that out of hours clinicians who lack access to previous medical and social histories appear to be admitting patients inappropriately [10,11]. This could be addressed by either encouraging patients to use routine services or by educating clinicians working in out of hours services. Previous studies have shown that patients vary in which services they call out during an acute illness, and this is influenced by patients' opinions on waiting times for appointments [19]. Improving access to routine services, improving the ability to consult specialist services during routine hours and improving the communication between these services could reduce use of out of hours services. They may also be confused about which service to call, which may mean that accessing the service with specialist knowledge is made more difficult for those with an LTNC [20]. The NSF (Quality requirement 11), states that when a person with a LTNC is admitted every effort should be made to consult specialist clinicians, in doing so they can provide support, information and training to generalist staff, ensuring the specific needs of such people can be met. An alert system, as discussed by the participants, is reported to be a relatively easy method of informing relevant clinicians and would appear to provide a valuable bridge between primary and secondary care, although, the capacity of clinicians to respond needs to be considered. Similarly, community clinicians who may not have immediate access to specialist advice could benefit if summaries of specialist consultations were to be retained with the patient and therefore readily available. Such interventions may also reduce the concerns admitting clinicians have about treating patients with specialist conditions in primary care.

Education of out of hours staff and general practitioners was felt to be important, and seen to be difficult in practice. Targeting GP's, as suggested, may help. In addition use of innovative methods of education may be of benefit, such as outreach visits that have been successfully used in education regarding dementia care or using a card playing teaching method as used in asthma management [21,22]. Factors that are known to affect the acceptability of educational interventions could be considered, such as demonstrating the connection with everyday practice, and being sensitive to the GP's perception of professional autonomy [23]. A gap in the understanding of current services was also shown by the participants themselves, for instance the neurological rehabilitation team based within Derby Hospitals, designed to work across primary and secondary care boundaries and to assist with the long term management of patients with LTNCs, and accessing condition specific workers employed by the voluntary sector (e.g. for motor neurone disease, Huntington's disease and muscular dystrophy) was not acknowledged by members.

There are several limitations to the study. Firstly, the optimal number of participants in a focus group is considered to fall between eight and 12, our study numbers are therefore relatively low [24]. When focus group numbers are low there is a risk that one or two participants will dominate conversation [24]. This appeared to be the case in the multi-disciplinary focus group where one member appeared to dominate discussions. However, involvement of a smaller group had the inadvertent benefit of allowing a more detailed, in-depth examination to take place, than might otherwise have been possible. Secondly, a representative of those working in the accident and emergency department was not included. Given the fact that this sector is central to many hospital admission procedures, this would have added further breadth to the study. Thirdly, the participants were selected through purposive sampling, this ensured that the samples were multi-disciplinary therefore presenting a variety of views. Random sampling of clinicians may have added to the generalisability of findings which may have been useful, however generalisability is not a primary aim of qualitative research.

In terms of future research or practice, in addition to that suggested earlier in this discussion, we would recommend that interventions which seek to improve communication between primary and secondary care services and, in particular, between generalist and specialist staff are explored. However, communication alone may not be sufficient, and studies that explore early assessment and intervention by specialist MDT services might also be considered.

We have explored two areas: the causes of inappropriate admissions and lengths of stay, as perceived by clinicians involved in the care of patients with LTNCs, and potential areas of intervention to reduce the occurrence of inappropriate admissions and/or LoS. Thorough documentation of the causes of inappropriate admissions and lengths of stay in this manner is unique and presents a systemic examination of the process from a variety of view points. Although explored in relation to people with LTNC's the findings are not at the level of treatment of neurological conditions but are more general in nature: access to specialist services, improved communication between primary and secondary care are all interventions that suggest these results may be relevant to non-neurological conditions.

## Conclusion

This study highlights a number of inter-related issues that are perceived to cause the inappropriate admission of patients with LTNCs to hospital. Structural causes, such as limitations in the provision of community health and social care resources, often prevent the optimal management of patients with LTNCs in the community. Limited knowledge regarding the management of patients with LTNCs and practical experience of managing such patients amongst non-specialist primary and secondary care clinicians can also be viewed as contributing factors. The transference of key information between clinicians in primary and secondary care and also between generalist and specialist clinicians, is thought to be sub-optimal and this can impede the management of patients with LTNCs.

Several areas of intervention were highlighted which could prevent inappropriate admissions and these included methods that seek to improve communication between clinicians. Specific suggestions of how this can be achieved included: (i) an alert system which would notify specialist clinicians when a patient under their care is admitted to hospital and (ii) a written medical summary of specialist consultations to be retained in the patient's home and therefore accessible to out of hours and generalist clinicians. Novel methods of educating clinicians such as outreach visits and card playing techniques might also improve the knowledge of LTNCs and their management amongst generalist clinicians.

## Competing interests

The authors declare that they have no competing interests.

## Authors' contributions

CLH carried out and designed the study and produced the first draft of the manuscript. MFP provided assistance with the design of the study and assisted with the revision of the manuscript. LP provided assistance with the design of the study and assisted with the revision of the manuscript.

## Pre-publication history

The pre-publication history for this paper can be accessed here:


